# Changes in chromatin state reveal ARNT2 at a node of a tumorigenic transcription factor signature driving glioblastoma cell aggressiveness

**DOI:** 10.1007/s00401-017-1783-x

**Published:** 2017-11-17

**Authors:** Alexandra Bogeas, Ghislaine Morvan-Dubois, Elias A. El-Habr, François-Xavier Lejeune, Matthieu Defrance, Ashwin Narayanan, Klaudia Kuranda, Fanny Burel-Vandenbos, Salwa Sayd, Virgile Delaunay, Luiz G. Dubois, Hugues Parrinello, Stéphanie Rialle, Sylvie Fabrega, Ahmed Idbaih, Jacques Haiech, Ivan Bièche, Thierry Virolle, Michele Goodhardt, Hervé Chneiweiss, Marie-Pierre Junier

**Affiliations:** 1 CNRS UMR8246, Inserm U1130, Neuroscience Paris Seine-IBPS, UPMC, Sorbonne Universités, Paris, France; 2 CNRS, UMR 8256, Laboratory of Neuronal Cell Biology and Pathology-IBPS, UPMC, Sorbonne Universités, Paris, France; 3 Interuniversity Institute of Bioinformatics in Brussels, Université Libre de Bruxelles, Brussels, Belgium; 4 UMRS-1126, Université Paris Diderot, Paris 7, Institut Universitaire d’Hématologie, EPHE, Paris, France; 5 CNRS UMR7277, INSERM U1091, Institut de Biologie Valrose, Université Nice-Sophia Antipolis, Université côte d’azur Nice, Nice, France; 6 Laboratoire Central d’Anatomie Pathologique, Hôpital Pasteur, Université Nice-Sophia Antipolis, Nice, France; 7 MGX-Montpellier GenomiX, c/o Institut de Génomique Fonctionnelle, Université Montpellier, Montpellier, France; 8 Plateforme Vecteurs Viraux et Transfert de Gènes, Université Paris Descartes-Structure Fédérative de Recherche Necker, INSERM US24/CNRS UMS3633, 75014 Paris, France; 9 Inserm U 1127, CNRS, UMR 7225, Sorbonne Universités, UPMC, UMR S 1127, Institut du Cerveau et de la Moelle épinière, ICM, 75013 Paris, France; 10 Laboratoire d’Excellence Medalis, Université de Strasbourg, CNRS, LIT UMR 7200, Strasbourg, France; 11 Pharmacogenomics Unit, Department of Genetics, Institut Curie Hospital, 75005 Paris, France

**Keywords:** Brain cancer, Glioma, Xenograft, ChIP

## Abstract

Although a growing body of evidence indicates that phenotypic plasticity exhibited by glioblastoma cells plays a central role in tumor development and post-therapy recurrence, the master drivers of their aggressiveness remain elusive. Here we mapped the changes in active (H3K4me3) and repressive (H3K27me3) histone modifications accompanying the repression of glioblastoma stem-like cells tumorigenicity. Genes with changing histone marks delineated a network of transcription factors related to cancerous behavior, stem state, and neural development, highlighting a previously unsuspected association between repression of *ARNT2* and loss of cell tumorigenicity. Immunohistochemistry confirmed ARNT2 expression in cell sub-populations within proliferative zones of patients’ glioblastoma. Decreased ARNT2 expression was consistently observed in non-tumorigenic glioblastoma cells, compared to tumorigenic cells. Moreover, *ARNT2* expression correlated with a tumorigenic molecular signature at both the tissue level within the tumor core and at the single cell level in the patients’ tumors. We found that *ARNT2* knockdown decreased the expression of *SOX9*, *POU3F2* and *OLIG2,* transcription factors implicated in glioblastoma cell tumorigenicity, and repressed glioblastoma stem-like cell tumorigenic properties in vivo. Our results reveal *ARNT2* as a pivotal component of the glioblastoma cell tumorigenic signature, located at a node of a transcription factor network controlling glioblastoma cell aggressiveness.

**Electronic supplementary material:**

The online version of this article (10.1007/s00401-017-1783-x) contains supplementary material, which is available to authorized users.

## Introduction

De novo glioblastoma, the most common and malignant primary brain tumor in adults, is a paradigmatic example of heterogeneous tumors [11, 49, 54, 64]. This heterogeneity stems from clonal selection of genomic and phenotypic variants, which arises not only from the accumulation of mutations but also from dynamic changes in cell states [27, 28]. As a result, cells with different functional properties co-exist such as proliferative versus non-proliferative, migratory versus static, stem-like versus non-stem, pro-angiogenic versus non-pro-angiogenic. Understanding the basis for this heterogeneity is of importance to efficiently target pivotal tumor cells, especially in glioblastoma that exhibits a dismal prognosis despite aggressive therapies.

Studies of glioblastoma cells endowed with stem-like and tumor-initiating properties (GBM stem-like cells) have shown that aside from the heterogeneity linked to distinct mutational loads, cancer cell diversification can be achieved at the functional level within an unchanged genomic background [16]. Glioblastoma cells have been shown to adopt distinct transcriptomic profiles combined with potentially distinct phenotypes and functional behaviors in response to environmental cues, which either favor acquisition of stem-like and tumorigenic properties [3, 24, 52] or in contrast induce their loss [41, 57]. Epigenetic plasticity has been shown to accompany GBM stem-like cell adaptations to their changing microenvironment [21, 22, 52, 71].

An important source of epigenetic plasticity is brought by post-translational histone modifications, such as methylation, acetylation, phosphorylation or ubiquitinylation of histone lysine (K) and arginine (R) residues [45]. These histone modifications alter either the affinity between DNA and histones or create binding sites for chromatin remodeling factors, thereby controlling DNA compaction and accessibility, subsequent transcription and hence ultimately functional outcomes [7, 65]. Pioneer studies in embryonic stem cells (ESC) first revealed the link between histone H3 K4 and K27 trimethylation (H3K4me3 and H3K27me3) with transcriptional expression and repression, respectively [50, 53, 78], the importance of which has been confirmed by large scales epigenomic studies notably in the brain [12]. In addition, these studies reported the existence of bivalent genes bearing both H3K4me3 and H3K27me3 histone marks in ESC [4, 6] as well as in adult multipotent/somatic stem cells [13, 51]. These bivalent genes are associated with RNA polymerase II at their transcription start sites and are thought to be in a “poised” state ready to be fully activated or repressed during differentiation [1, 10, 37, 50].

**Fig. 1:**
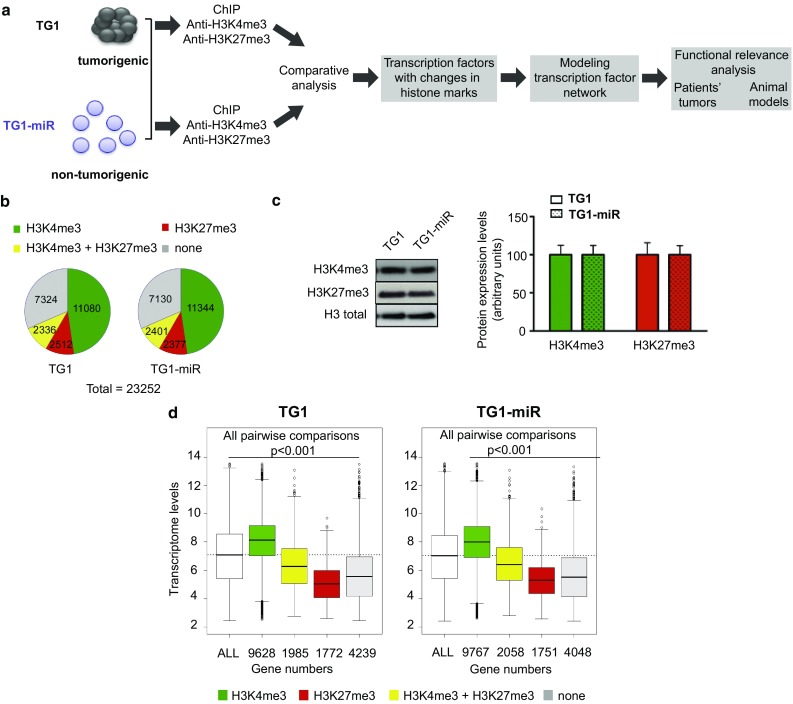
Global maintenance of histone marks in differentiated GBM stem-like cells. **a** Schematic overview of the strategy of the study. See text for details. **b** Global distribution of histone marks is similar across the genome in TG1 and in TG1-miR (TG1 overexpressing miR-302–367 cluster). None = genes non-detected following ChIP-seq analysis with H3K4me3 or H3K27me3 antibodies. Their numbers were calculated using the human reference genome hg19. **c** miR-302–367 expression does not change the overall proportion of H3 bearing a trimethylation of K4 or K27. Left panel: example of Western blot detection of H3K4me3 and H3K27me3. Right panel: densitometry analysis of relative levels of H3K4me3 and H3K27me3 forms normalized to the total levels of H3. Mean ± SD, *n* = 4 independent experiments. **d** Positive correlation between chromatin state changes and gene expression levels in TG1 and TG1-miR determined with DNA microarrays. Box plots show the level of transcripts according to the histone marks associated to the corresponding gene. White boxes: all genes regardless of the histone mark (ALL). Green boxes: genes associated with the active mark H3K4me3. Yellow boxes: genes associated with both marks (bivalent mark). Red boxes: genes associated with the repressive mark H3K27me3. Gray boxes: genes non associated with either histone mark (none). The dotted line represents the median level of all genes analyzed (white box). All pairwise differences among group means are statistically significant (*p* < 0.001, pairwise *t* test)

Here, we focused on the H3K4me3 and H3K27me3 marks to gain insights into the transcription factor network that sustains glioblastoma cell tumorigenic properties through a bottom-up approach schematized in Fig. 1a. We used as a starting paradigm human glioblastoma cells expressing or not expressing the micro-RNA cluster miR-302–367. Indeed our previous studies have shown that the expression of miR-302–367 represses the stem-like, and most importantly, tumor-initiating properties of human glioblastoma cells [22]. Mapping the genes epigenetically modified in glioblastoma cells following repression of their tumorigenic properties, allowed the modeling of an interrelated array of transcription factors implicated in pathways important for malignancy, stem cell state, and neural development. Most importantly, our results pinpointed a previously unsuspected involvement of the hypoxia-inducing factor (HIF) family member aryl hydrocarbon receptor nuclear translocator 2 (ARNT2) in the control of glioblastoma cell aggressiveness. We then verified and extended our findings using a combination of bioinformatics analysis of independent glioblastoma datasets, analysis of patients’ tumor tissues, genetic manipulations of independent additional glioblastoma cell cultures and in vivo experiments. Our results demonstrate that ARNT2 controls the expression of several transcription factors associated with the stem-like properties of glioblastoma cells, and is essential for full tumorigenicity of glioblastoma cells.

## Materials and methods

### Cell cultures

GBM stem-like cells with mesenchymal (TG1), and classical transcriptome profiles (6240** and 5706**) were isolated from neurosurgical biopsy samples of human primary glioblastoma affecting 62–68-year-old patients, with a IDH wild-type status, and characterized for their stem-like and tumor-initiating properties as described [2, 25, 56, 62, 63, 67]. TG1-miR was derived from TG1 as described [22]. GBM stem-like cells 6240** and 5706** were stably transduced with a lentiviral construct encoding the firefly luciferase (6240**) or the firefly luciferase and the fluorescent protein GFP (5706**) [62]. All cells were cultured in defined medium containing bFGF and EGF. TG1, 6240**, and 5706** stem-like cells were transduced with lentiviral vectors encoding a control or an ARNT2 shRNA construct (pLKO.1-HPGK-puro-U6-non mammalian shRNA control, and pLKO.1-HPGK-puro-CMV-TGFP-U6-shARNT2, Sigma, France). All non-transduced cells were eliminated following puromycin treatment (2 µg/ml) for 10 days. Lentivirus was produced by the Plateforme vecteurs viraux et transfert de gènes (Necker Federative structure of research, University Paris Descartes, France).

### Viable cell counting

Trypan blue exclusion test was used to determine the numbers of viable cells (Trypan blue solution, ThermoFisher, 0.4% v/v, 3 min incubation at room temperature). Blue and white cells (dead and alive, respectively) were counted with the Countess automated cell counter (Thermo Fisher, France).

### Extreme limiting dilution assays (ELDA)

Cells were plated in 96-well plates at 1, 5, and 10 cells/well/100 μl as previously described [2]. The percentage of wells with cell spheres was determined after 7 days. The analysis of the frequency of sphere-forming cells, a surrogate property of brain cancer stem-like cells [24] was performed with software available at http://bioinf.wehi.edu.au/software/elda/ [34].

### ChIP-seq sample preparation and analysis

ChIP assays were performed using ChIP-IT Express Magnetic Chromatin Immunoprecipitation kit following the manufacturer’s protocol (Active motif, France) and 2 × 10^6^ cells per sample and per epitope. Briefly, TG1 and TG1-miR-302–367 cells were cross-linked in 0.5% formaldehyde/PBS for 10 min at room temperature and then treated with 0.125 M glycine in PBS pH 7.4 for 5 min at room temperature. Samples were subsequently washed twice with ice-cold PBS and once with ice-cold PBS supplemented with protease inhibitors cocktail prior to be lysed. Chromatin fragments ranging from 200 to 500 bp were obtained by sonication (10 pulses at 40% of amplitude, 20 s ON, 50 s OFF, Sonics Vibracell VCX 130 sonicator, Sonics and materials, USA). Chromatin was then incubated overnight at 4  °C on a rotor with anti-H3K4me3 (Millipore, 07-473, France) or anti-H3K27me3 (Millipore, 07-449, France). The chromatin–antibody complexes were then washed, eluted and reverse cross-linked at 65 °C for 5 h. The eluted DNA was treated sequentially with Proteinase K and RNase A, and purified with the MinElute Reaction Cleanup Kit (Qiagen, 28204, France). The amount of DNA obtained was measured with a Qubit fluorometer (ThermoFisher, France). Library preparation was performed using the ChIP-Seq Sample Preparation kit (Illumina) on 10 ng of purified ChIP DNA samples. Libraries were sequenced on a Hiseq 2000, 1 library per lane, following standard procedures (Sequencing Platform of Montpellier GenomiX, MGX, France). An input control was sequenced for each cell type, and used for normalization. Alignments of the reads to the hg19 human reference genome were performed with CASAVA (1.8.2 version, Illumina). Alignments with more than two mismatching bases within the 32 first bases of the read were discarded. Visualization was performed with the Integrative Genomics Viewer (www.broadinstitute.org/igv/home). Peak detection was performed using the MACS software version 1.4.2 (http://liulab.dfci.harvard.edu/MACS/) [76] with input control libraries from the corresponding cell types. Peaks were then annotated using a window of ± 20 kb with respect to the coordinates of the beginning and end of RefSeq transcripts. More than 150 million short reads were obtained for all samples. These short reads were uniquely aligned to the human genome, resulting in a 77 and 76% of the genome covered in TG1 and TG1-miR, respectively. The data have been deposited in NCBI’s Gene Expression Omnibus [20] and are accessible through GEO Series accession number GSE98330 (https://www.ncbi.nlm.nih.gov/geo/query/acc.cgi?acc=GSE98330). ARNT2 ChIP was performed as described above using anti-ARNT2 antibodies (Santa Cruz, Cliniscience sc-5581X, France) and 100–1000 bp 5706** chromatin fragments. QPCR analysis was performed on total (input) and immunoprecipitated chromatin, and results normalized over the corresponding input signal. Enhanced representation of the regions of interest was compared to *TBP* promoter negative control. Sequences of all primers used for ChIP-qPCR are listed in Online Resource 1.

### Gene expression analysis

Total RNA was prepared using the RNeasy Plus Universal kit (Qiagen, France) according to the manufacturer’s instruction. An on-column DNase digestion was performed during the extraction to yield a pure RNA fraction (RNase-Free DNase Set, Qiagen). cDNA was prepared using the QuantiTect Reverse Transcription Kit (Qiagen) according to manufacturer’s instructions. Expression profiles of TG1 and TG1-miR-302–367 were determined using Affymetrix 1.0 Human Exon ST arrays according to the manufacturer’s instructions in three successive cell passages (Strasbourg France Génomique platform, France). The signals obtained were normalized to a series of housekeeping genes (30 in total), and log2 transformed. RT-QPCR assays were performed using a Quantstudio6 (Applied Biosystems, France). PCR was performed using the SYBR Green PCR Core Reagents kit (Applied Biosystems, France). The thermal cycling conditions comprised an initial denaturation step at 95 °C for 10 min and 45 cycles at 95 °C for 15 s and 60 °C for 1 min. Transcripts of the TBP gene encoding the TATA box-binding protein (a component of the DNA- binding protein complex TFIID) were quantified as an endogenous RNA control. Quantitative values were obtained from the cycle number (Ct value), according to the manufacturer’s manuals (Applied Biosystems). Sequences of all primers used for QPCR are listed in Online Resource 2.

### Expression profiling

Statistical and graphical analyses of ChIP-seq and microarray data were performed using the R software version 3.2.3 (http://cran.r-project.org/). Gene ontology (GO) analysis was performed with DAVID software (version 6.8, http://david.abcc.ncifcrf.gov/). GO analysis of all genes changing histone marks in TG1-miR compared to TG1 was achieved using all human genes as background (Homo Sapiens from DAVID). GO analyses of genes exchanging an active for a repressive histone mark and vice versa between TG1 and TG1-miR were achieved using as background all the genes with differing histone marks in TG1-miR and TG1. Genes encoding transcription factors were retrieved using the KEGG (http://www.genome.jp/kegg/), and Genomatix databases (Genomatix, Germany). Interactions between the retrieved set of 202 transcription factors were analyzed with the STRING database (version 10.0, http://string-db.org/). Heat maps and *z* scores were downloaded from the IVY dataset (http://glioblastoma.alleninstitute.org), and analyzed with XLSTAT version 1.2. z-score graphs were generated with Prism 6.0 software (GraphPad). ARNT2 mRNA expression was analyzed using publicly available data using the R2 Genomics Analysis and Visualization Platform (http://r2.amc.nl) (Lee, mixed glioblastoma dataset, GEO ID: GSE4536) and the HGGC website (http://130.238.55.17/hgcc/). TCGA transcriptome dataset of 481 surgical tissue samples of untreated primary glioblastoma (tcga 540 glioblastoma) was analyzed using the R2 Genomics Analysis and Visualization Platform. Single glioblastoma cell transcriptomes were obtained at https://www.ncbi.nlm.nih.gov/geo/query/acc.cgi?acc=GSE57872, and analyzed with XLSTAT version 1.2.

### Immunoblotting

Cells were harvested, washed with PBS and cell lysis was performed in 50 mM Tris–HCl pH 7.4 buffer containing 1% Triton X-100, 150 mM NaCl, 0.5 mM EGTA, 0,5 mM EDTA and anti-protease cocktail (Complete Protease inhibitor Cocktail Tablets, Roche, France). Protein extracts (30 μg) were separated by SDS-PAGE and transferred to Hybond-C Extra nitrocellulose membranes (GE Healthcare, USA) as described [70]. The following antibodies were used for immunoblotting: anti-actin (Millipore Chemicon, 1:10000), anti-ARNT2 (Santa Cruz, 1:2000), anti-histone H3 (Abcam, 1:50000), anti-trimethyl-histone H3 (Lys 4) (Cosmobio, 1:500), and anti-trimethyl-histone H3 (Lys 27) (Upstate-Millipore, 1:3000). The secondary antibodies were anti-mouse IgG (Santa Cruz Biotechnology, 1:10000) and anti-rabbit IgG (GE Healthcare, 1:10000). Signal detection was performed with the ECL + chemiluminescence detection system (PerkinElmer, France). Densitometric analysis was achieved using ImageJ software.

### Immunohistochemistry

Morphologic examination of patients’ glioblastoma resections was performed on Hematoxylin and Eosin stained sections (3–4 μm). Immunolabeling was performed using an automated system (Autostainer Dako, Glostrup Denmark). Deparaffinization, rehydration and antigen retrieval were performed using the pretreatment module PTlink (Dako). ARNT2 immunostaining was achieved using anti-ARNT2 (Santa Cruz, 1:50) and anti-Ki67 antibodies (MIB-1, Dako, prediluted). Immunostaining was scored by a pathologist (FBV).

Xenografted mouse brains were dissected after killing of the mice at 45 days post-graft of 6240** or 42 days post-graft of 5706** GBM stem-like cells expressing shControl or sh*ARNT2*. The brains were fixed in 4% paraformaldehyde in PBS for 48 h at 4 °C, cryoprotected in 30% sucrose in PBS at 4 °C until the tissue sank, frozen in isopentane at – 40 °C, and stored at – 80 °C. Cryostat sections of 30-µm-thickness were cut in the frontal plane. Thirteen sections, from the olfactory bubs to the posterior end of cerebellum were selected for the analysis. Sections were incubated with DAPI (Sigma, France) for 10 min at room temperature. Sections staining was analyzed with a fluorescent microscope (Axioplan 2, Zeiss). Images were acquired on digital camera (DXM 1200, Nikon, USA) using Zen 2 software (Zeiss) and prepared using Adobe Photoshop software (Adobe Systems, San José, USA).

### Intracranial xenografts

The animal maintenance, handling, surveillance, and experimentation were performed in accordance with and approval from the Comité d’éthique en expérimentation animale Charles Darwin No. 5 (Protocol #3113). 6240** and 5706** GBM stem-like cells transduced with a luciferase encoding lentivirus and either a shControl or a sh*ARNT2*, were used. 140,000 (6240** and 5706**), 40,000 (6240**, 5706**), 20,000 (6240**) or 10,000 (6240**) cells were injected stereotaxically into the striatum of anesthetized 8- to 9-week-old nude mice (Envigo Laboratories, France), using the following coordinates: 0 mm posterior and 2.5 mm lateral to the bregma, and 3 mm deep with respect to the surface of the skull. Luminescent imaging was performed on a photonImager Biospace (Biospace Lab, France), after intra-peritoneal injection of 150 μl luciferin 20 mM (Thermo Fisher, 88293). Tumor formation was monitored by bioluminescence until all mice of the control group showed a signal. Bioluminescent signals were visualized with M3 Vision software (Biospacelab).

### Statistical analysis

R version 3.2.3, XLSTAT version 1.2 or Prism 6.0 software (GraphPad) were used for statistical analyses. The level of significance was set at *p* < 0.05. The type of statistical test used is provided in the figure legends. All experiments were performed using independent biological samples with the exception of the ChIP-seq. All experiments were repeated at least three times in an independent manner with the exception of the microarray experiment. PCA analysis was performed on XLSTAT version 1.2, based on a Pearson correlation matrix. First and second component (F1 and F2 axis) were used to generate a correlation circle where the variables (genes) were plotted as vectors according to their correlation with F1 and F2 axis.

The figures were prepared using Adobe Illustrator (Adobe Systems).

## Results

### Repression of GBM stem-like cell properties is accompanied by discrete changes in epigenetic profiles

Lentiviral expression of miR-302–367 in the TG1 human GBM stem-like cell line (referred to as TG1-miR) resulted in loss of their stem-like and tumorigenic properties [22]. H3K4me3 and H3K27me3 profiling of TG1 and TG1-miR was performed by ChIP followed by deep sequencing (data accessible at https://www.ncbi.nlm.nih.gov/geo/query/acc.cgi?acc=GSE98330). For each cell type analyzed, approximately 16,000 genes (~ 70% of the complete human exome) were found to be associated with the H3K4me3 and/or H3K27me3 mark (Online Resource 3). This analysis revealed a predominance of genes (~ 48%) associated with the active H3K4me3 mark in TG1 and in TG1-miR (Fig. 1b). Only ~ 10% were associated with the repressive H3K27me3 mark. An equivalent proportion (~ 10%) was associated with the bivalent mark (H3K4me3 and H3K27me3) (Fig. 1b). Western blot assays further demonstrated similar H3K27me3 and H3K4me3 protein levels in TG1 and TG1-miR (Fig. 1c). As described in other cell types [5, 78], both marks were enriched in TG1 and TG1-miR at the level of the TSS, with the H3K27me3 mark being in addition spread along the gene bodies (Online Resource 4). Furthermore, as expected, the highest transcript levels were observed in the group of genes associated with the H3K4me3 mark, the lowest transcript levels in the group of genes associated with the H3K27me3 mark, whereas genes associated with the bivalent mark had intermediate expression levels (Fig. 1d). The mean transcript level of the group of genes associated with none of the marks was slightly above the mean expression level of the genes carrying the H3K27me3 mark, suggesting that these genes tended to be repressed. Altogether, these results show that miR-302–367 does not alter global levels of H3K4me3 and H3K27me3, or the proportion of genes associated with either modification or the repartition of the histone marks along the genes.

**Fig. 2:**
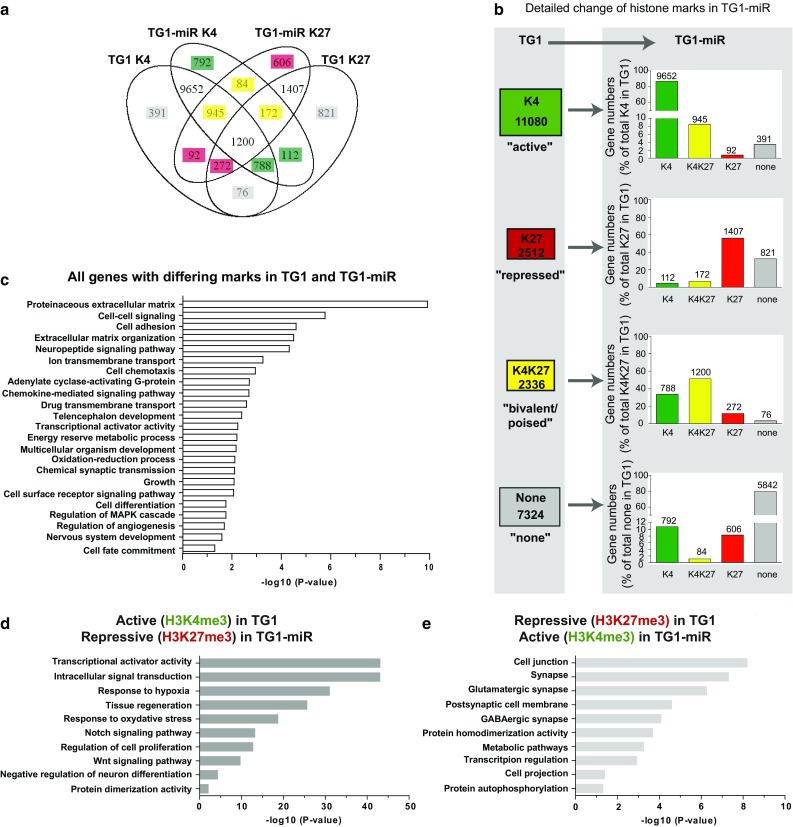
Loss of tumorigenic properties is associated with rearrangement of H3K4me3 and H3K27me3 marks in discrete subsets of genes. **a** Venn diagram illustrating genes groups with differing histone marks in TG1 and TG1-miR. Numbers of genes in each category are indicated. Numbers highlighted with colors correspond to gene with marks changing following the expression of miR-302–367, colors representing the final epigenetic status. K4 = K4me3. K27 = K27me3. **b** Detailed representation of histone mark changes observed in the TG1-miR. K4 = K4me3. K27 = K27me3. **c** Gene ontology analysis of all genes with changes in H3K4me3 and H3K27me3 marks between TG1 and TG1-miR. **d** Gene ontology analysis of genes undergoing a transition from H3K4me3 in TG1 to H3K27me3 in TG1-miR. **e** Gene ontology analysis of genes undergoing a transition from H3K27me3 in TG1 to H3K4me3 in TG1-miR

While the proportion of genes associated with each histone mark was globally unchanged, further analysis revealed a set of 5151 genes exhibiting a change in histone modifications between TG1 and TG1-miR. This number corresponded to 22% of the total number of sequenced genes. The overlap of genes differentially expressed between TG1 and TG1-miR is depicted with a Venn diagram (Fig. 2a). Detailed analysis pointed to the H3K4me3 mark as the most conserved mark following miR-302–367-induced repression of the cells’ properties (Fig. 2b, Online Resource 3). Of the 11,080 genes enriched for H3K4me3 mark in TG1, only 92 (~ 0.8%) switched to H3K27me3, whereas 945 (~ 8.5%) acquired a bivalent chromatin state and 391 (~ 3.5%) lost the mark. Of the 2512 genes associated with H3K27me3, 112 genes (~ 4.5%) switched to H3K4me3 marks. Of the 2336 bivalent genes, 788 (34%) turned into H3K4me3 only, whereas 272 (~ 11.5%) retained only the H3K27me3 mark. In summary, close to half of the repressed (H3K27me3, 44%) and poised (bivalent, 49%) genes underwent a change in their epigenetic marks, whereas only a minority of active genes (H3K4me3, 13%) underwent epigenetic modifications in TG1-miR. Altogether, these results show that the repressive effects of miR-302–367 are accompanied by changes in the chromatin state of a subset of genes while the proportional repartition of each histone mark across the genome is conserved.

### Changes in histone modifications highlights ARNT2 as a core member of a transcription factor network associated with maintenance of GBM stem-like cell properties

To identify the function of the genes whose chromatin state is modified following repression of the properties of GBM stem-like cells, we performed functional enrichment analysis using DAVID toolbox [14, 15]. In a first step, we performed a gene ontology (GO) analysis using the whole set of the 5151 genes associated with different histone modifications in TG1 and TG1-miR (Fig. 2c, Online Resource 5). Several terms related to the central nervous system were significantly enriched as expected for cells derived from the brain. Consistent with the drastic change in cell functional state induced by the miR-302–367 cluster [22], we also found terms grouping genes located at the core of cell behavior (such as transcription, metabolism), and related to development and differentiation. We also found categories associated with cell motility (cell adhesion, differentiation, and chemotaxis) consistent with the propensity of TG1-miR to adhere to a permissive plastic support and with the loss of their invasive capacity [22]. Functional enrichment analysis restricted to genes that permute from an active to a repressive histone mark showed enrichments in terms related to the maintenance of the undifferentiated features of the cells (Notch and Wnt signaling pathways, negative regulation of neuron differentiation, Fig. 2d, Online Resource 5). Conversely, genes that changed from the repressive H3K27me3 to the active H3K4me3 mark showed enrichments in terms related to neural cell differentiation (Fig. 2e, Online Resource 5).

**Fig. 3:**
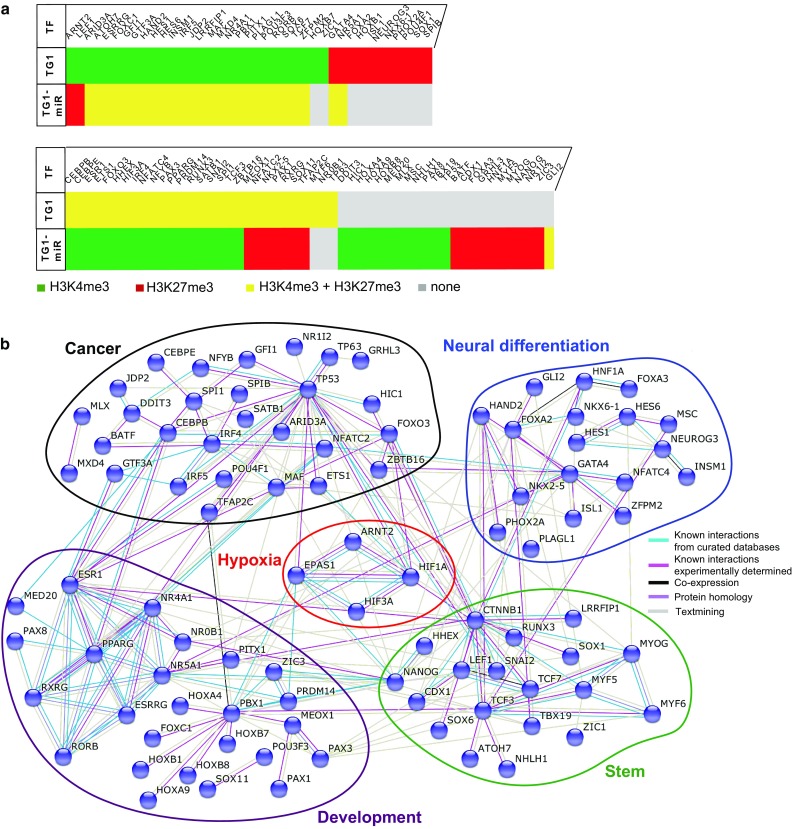
Epigenetic regulation of transcription factors highlights ARNT2 as a new actor in the maintenance of GBM stem-like cells properties. **a** Overview of the transcription factors from the STRING network undergoing transition in epigenetic marks in TG1-miR. **b** STRING analysis of transcription factors changing H3K4me3 and H3K27me3 marks in TG1-miR. Edges between proteins symbolize the confidence index of the interaction probability. Edges are colored based on the source of information: known interactions from curated databases (light blue), known interactions experimentally determined (pink), co-expression (black), protein homology (purple) and text mining (gray). Note that HIF1A, EPAS1 (HIF2A) and CTNNB1 (beta-catenin) have the same histone mark (H3K4me3) in TG1 and TG1-miR

**Fig. 4:**
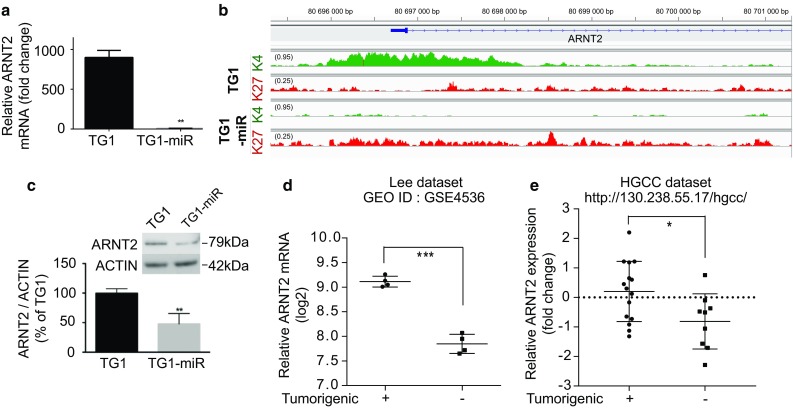
Decreased ARNT2 expression is associated with non-tumorigenic glioblastoma cells. **a** Decreased *ARNT2* mRNA levels in TG1-miR compared to TG1. QPCR assay. **p < 0.01, unpaired *t* test with Welch’s correction, mean ± SD, *n* = 3 independent biological samples. **b** Loss of the active H3K4me3 mark and gain of the repressive H3K27me3 mark around the *ARNT2* transcription start site in TG1-miR. **c** Decreased ARNT2 protein levels in TG1-miR compared to TG1. Western blot analysis. **p < 0.01, unpaired *t* test with Welch’s correction, mean ± SD, *n* = 3 independent biological samples. **d** Analysis of published transcriptome dataset of early passage (P3) glioblastoma cells isolated from four human tumors, either endowed with self-renewing and tumor-initiating properties or devoid of them following serum-treatment [41]. ***p < 0.001, unpaired *t* test with Welch’s correction, mean ± SD, *n* = 4. **e** Analysis of the publicly available HGCC transcriptome dataset of glioblastoma cells isolated from distinct patients’ tumors and characterized for their ability to initiate tumors [73]. *p < 0.05, unpaired *t* test with Welch’s correction, mean ± SD, *n* = 15 (Tumorigenic), *n* = 9 (non-Tumorigenic)

TG1 cells overexpressing miR-302–367 cluster exhibit a drastic change in their functional state, from tumorigenic cells with stem cell-like features to non-tumorigenic cells lacking stem-like properties [22]. To further decipher the molecular basis of this phenotypic change, we focused our analysis on transcription factors, which are likely to play a pivotal role in driving the changes in functional state. Genes encoding transcription factors were retrieved using the KEGG (http://www.genome.jp/kegg/), and Genomatix databases (Genomatix, Germany). Interactions between the set of 202 transcription factors retrieved from the 5151 genes presenting a change in epigenetic mark (Online Resource 6) were then analyzed using the STRING software (http://string-db.org/ version 10.0, [69]. Only interactions with a high confidence level (0.7) were selected. Addition of three supplementary transcription factors, which were associated with the active H3K4me3 mark in both TG1 and TG1-miR (hypoxia inducible factor 1α/*HIF1A*, hypoxia inducible factor 2α/*EPAS1* and beta-catenin/*CTNNB1*), allowed optimization of the modeling of a network including a maximal number of elements. This analysis yielded a densely connected network gathering 91 transcription factors whose genes are associated with changing histone modifications following expression of the miR-302–367 cluster (Fig. 3a and b). The network included five nodes grouping transcription factors not only involved in cancer, but also in stemness (e.g., NANOG, LEF1), in neural differentiation (e.g., FOXA2/3, NKXs, NEUROG3) and in development (e.g., HOXs, PAXs) that could all be related to a node regrouping transcription factors of the hypoxia pathway. Interestingly, this network included two of the three transcription factors that exchanged the active H3K4me3 mark for the repressive H3K27me3 mark, namely LEF1 and ARNT2. LEF1 is a key component of the Wnt/β-catenin signaling pathway, a pathway known to contribute to the maintenance of the properties of GBM stem-like cells [38, 77, 79]. ARNT2 is considered, like its paralog ARNT, as an accessory partner required for full transcriptional activity of several proteins including HIF1α, HIF2α, AHR, NPAS4, and SIM1 [17, 29, 61, 66]. Analysis of mRNA levels showed that *ARNT2* was the only transcription factor in the hypoxia pathway (ARNT, ARNT2, HIF1α/*HIF1A*, HIF2α/*EPAS1*, HIF3α/*HIF3A*, and HIF1α inhibitor/*HIF1AN*), to exhibit a drastic reduction of its mRNA levels in TG1-miR compared to TG1 (Fig. 4a, Online Resource 7). This finding was coherent with changes in histone modifications at the *ARNT2* locus from H3K4me3 in TG1 to H3K27me3 in TG1-miR (Fig. 4b). We did not find miR-302–367 target sites within the *ARNT2* mRNA (MIRBase, http://www.mirbase.org/), indicating that decreased *ARNT2* expression in TG1-miR does not stem from direct targeting by miR-302–367. Immunoblot analysis showed that reduced transcription of *ARNT2* was associated with a decrease in ARNT2 protein levels (Fig. 4c). These results together with the scant information currently available on the role of ARNT2 in cancer, led us to investigate further the possible implication of ARNT2 in the regulation of glioblastoma cell properties.

### ARNT2 is functionally associated with a molecular signature linked to glioblastoma cell tumorigenicity within the patients’ tumors

We first analyzed *ARNT2* expression in two published independent transcriptome datasets of glioblastoma cells either devoid of or endowed with tumor-initiating properties [41, 73]. In agreement with our observations, we found that ARNT2 expression was downregulated in non-tumorigenic cells compared to tumorigenic cells in both datasets (Fig. 4d, e). Further, the analysis of the TCGA transcriptome dataset of 481 surgical tissue samples of untreated primary glioblastoma using the GlioVis Platform [9] showed lower *ARNT2* mRNA levels in glioblastoma tissues than in non-tumoral brain tissues (Online Resource 8A). The finding of higher ARNT2 mRNA levels in normal brain tissues than in GBM tissues from which neurons are absent is coherent with the high ARNT2 expression in mature neurons [18, 19, 32]. Analysis of the TCGA glioblastoma dataset and the French glioma dataset gse16011 [26], showed no variation in ARNT2 expression according to *MGMT* status, *IDH1* mutation or *EGFR* amplification (not shown). In accordance with the narrow distribution of ARNT2 expression levels across glioblastoma samples (Online Resource 8A), no correlation could be disclosed using the R2 Genomics Analysis and Visualization Platform (http://r2.amc.nl) between variations in ARNT2 mRNA levels and the overall survival of patients (Online Resource 8B).

**Fig. 5:**
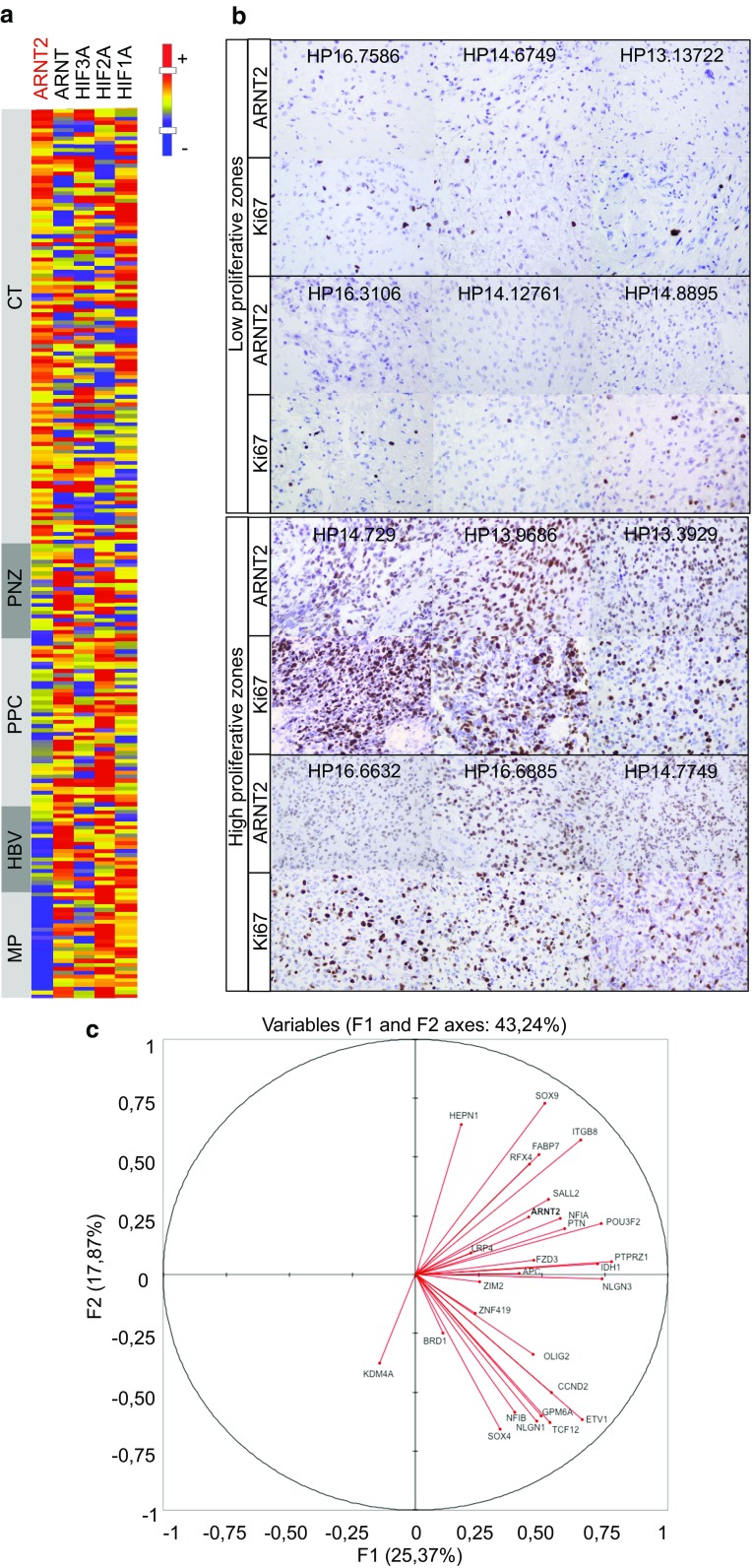
ARNT2 is expressed in patients’ glioblastoma and is associated with a tumorigenic/stem signature. **a** ARNT2 expression prevails in glioblastoma areas of high tumor cell density (CT). Note the absence of clear-cut correlation between ARNT2 and other HIF family members in glioblastoma. Heat map representation of mRNA levels evaluated in distinct glioblastoma zones (IVY dataset). Glioblastoma zones defined according to IVY gap white paper (May 2015 v.1) as follows. CT: cellular tumor zone of glioblastoma constituting the major part of core, with 100/1 to 500/1 tumor cell to normal cell ratio. PNZ: perinecrotic zone corresponding to a 10-30 cells’ boundary along a necrotic zone but lacking a clear demarcation. PPC: pseudopalisading cells aggregates around a necrotic area. HBV: hyperplastic blood vessels with thickened walls and endothelial cell proliferation. MP: areas of microvascular proliferation characterized by two or more vessels sharing a common wall. **b** ARNT2 positive cells are enriched in high proliferative zones of the tumor, as shown by immunohistochemical staining of ARNT2 and Ki67 in sister sections of patients’ glioblastoma. Magnification ×400. **c** ARNT2 expression in patients’ glioblastoma tumor core correlates with expression of genes composing the glioblastoma stem-like cells signature (listed in Online Resource 6). Correlation circle with F1 and F2 principal components. Analysis performed with transcriptome data from IVY dataset glioblastoma core zones (http://glioblastoma.alleninstitute.org)

Glioblastoma are characterized by the intermingling of differing tumor tissues including dense tumor areas where cancer cells predominate (“cellular tumor areas”), necrotic and perinecrotic areas with sparser tumor cells, areas more or less angiogenic, and infiltrated areas where tumor cells are distributed through the brain parenchyma. To refine the analysis of ARNT2 and its family kin in glioblastoma, we explored its expression using the IVY dataset, which provides gene mRNA levels in distinct glioblastoma zones (http://glioblastoma.alleninstitute.org). The results of this analysis singled out ARNT2 among the other HIF family members. *ARNT2* mRNA levels were higher in the cellular areas of the tumors than in the perinecrotic zones and barely detectable in tumor blood vessels. *HIF3A* expression was evenly distributed in the different tumor zones, while *HIF1A, HIF2A,* and *ARNT* expressions were enriched in the perinecrotic zones and/or in blood vessels of the tumor (Fig. 5a, Online Resource 9). Coherent with the profile of *ARNT2* mRNA distribution across glioblastoma areas, immunohistochemical analysis of neurosurgical samples of patient’s tumors revealed enrichment in ARNT2-expressing cells with increased distance from necrosis (Online Resource 9B). Importantly, this analysis revealed that ARNT2-expressing cells were especially enriched in the proliferative zones of the tumor (Fig. 5b).

To further explore the relevance of ARNT2 expression in the context of the human tumors, we compared its expression in the tumor core areas of glioblastomas (IVY dataset) with the expression of genes associated with glioblastoma cells endowed with tumorigenic and stem-like properties. We used a 28 molecules’ signature delineated by the Bernstein laboratory from the combined analysis of single cell transcriptome profiles of cultured GBM stem-like cells and of 254 cells sampled from five different glioblastomas [55]. We retrieved expression data from 27 of the 28 components of the signature from the IVY dataset (Online Resource 10). Principal component analysis (PCA) showed that *ARNT2* expression co-varied with the tumorigenic/stem signature along the first principal component axis (F1 axis) (Fig. 5c). We verified whether this co-variation occurred also at the single cell level using the published transcriptome profiles of 254 glioblastoma cells [55]. This dataset contains 25 of the 28 signature’s components (Online Resource 10). Principal component analysis showed that *ARNT2* expression co-varied also with the stem signature at the single cell level (Online Resource 11).

**Fig. 6:**
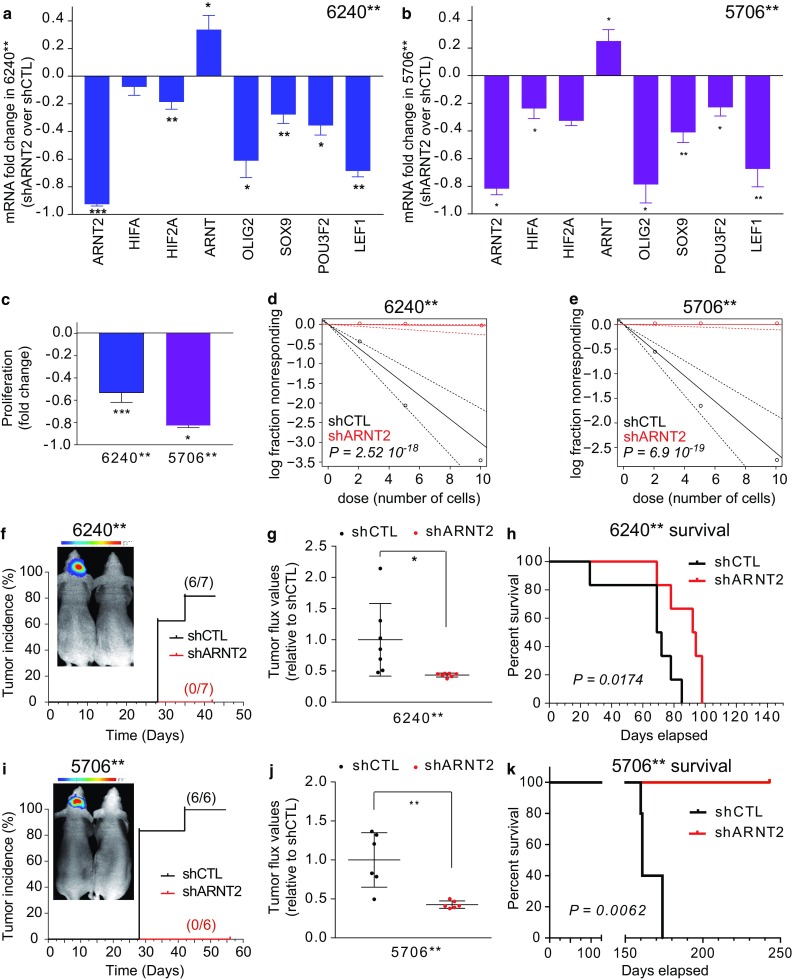
ARNT2 down-regulation impairs tumor initiation and development. **a**, **b** Consequences of ARNT2 down-regulation on the expression of HIF family members (HIF1A, HIF2A, ARNT), on core components of the tumorigenic/stem signature of glioblastoma cells (OLIG2, SOX9, POU3F2), and on the effector of the Wnt signaling pathway, LEF1. QPCR assay. Results are presented as fold changes in mRNA levels detected in GBM stem-like cells expressing sh*ARNT2* compared to shControl (shCTL). *p < 0.05, **p < 0.01, ***p < 0.001, unpaired *t* test with Welch’s correction, mean ± SD, *n* = 3 independent biological samples. **c** Down-regulation of *ARNT2* is accompanied with decreased cell proliferation. sh*ARNT2* versus shControl. *p < 0.05, ***p < 0.001, unpaired *t* test with Welch’s correction, mean ± SD, *n* = 3 independent biological samples. **d**, **e** Knocking-down of ARNT2 inhibits the sphere‐forming capability of GBM stem-like cells. Extreme limiting dilution assays. Sphere formation was scored 7 days after seeding 6240** (**d**) and 5706** (**e**) GBM stem-like cells expressing shControl or shARNT2. Frequency of sphere‐forming cells: 6240** shCTL = 1/3.32 (lower 4.68, upper 2.41); 6240** shARNT2 1/267.47 (lower 1887.52, upper 38.27), *n* = 16, p = 2.52 10^−18^. 5706** shCTL = 1/3.84 (lower 5.42, upper 2.77); 5706** shARNT2 1/Inf (lower Inf, upper 91.30), *n* = 16, p = 6.9 10^−19^. **f**, **i** Knocking-down of ARNT2 inhibits tumor incidence. Bioluminescent analyses of tumor growth initiated by grafting 6240** (**f**) and 5706** (**i**) GBM stem-like cells transduced with a luciferase construct and either a shControl or a sh*ARNT2* construct. The percentage of tumor incidence was monitored for 6 (6240**) and 8 (5706**) weeks. **g**, **j** Bioluminescent analyses of tumor growth initiated by grafting 6240** (**g**) and 5706** GBM stem-like cells transduced with a luciferase construct and either a shControl or a sh*ARNT2* construct. 28 days post-graft. Quantification of the bioluminescent signals. Mean ± SD, *n* = 7 mice per group for 6240** (**g**) and *n* = 6 per group for 5706** (**j**). **h**, **k** Kaplan–Meier survival curves demonstrating a significant survival benefit of mice grafted with GBM stem-like cells expressing shARNT2 compared to mice grafted with GBM stem-like cell expressing shControl. 6240** shCTL and shARNT2, each *n* = 6 (**h**). 5706*** shCTL, *n* = 5, 5706** shARNT2, *n* = 4 (**k**). Log-rank Mantel–Cox test

To probe the functional relevance of the co-variations disclosed by analysis of glioblastoma tissues and single cells transcriptomes, we determined the effect of *ARNT2* knockdown on the expression of *SOX9*, *POU3F2* and *OLIG2* in independent GBM stem-like cell cultures (6240** and 5706**) distinct from TG1 and TG1-miR. These three genes were selected with respect to their previously demonstrated role in glioblastoma cell tumorigenicity [31, 44, 68]. An 80 to 90% decrease in *ARNT2* mRNA level was observed in cells expressing sh*ARNT2* (Fig. 6a, b, Online Resource 13). We observed decreased *SOX9*, *POU3F2* and *OLIG2* mRNA levels following ARNT2 knockdown using lentiviral transduction of small hairpin RNA (shControl or sh*ARNT2*, Fig. 6a, b, Online Resource 12). Similar results were obtained in TG1 cells expressing sh*ARNT2* (Online Resource13). In addition, we verified that *ARNT2, OLIG2, POU3F2* and *SOX9* expressions did not change in conditions of reduced oxygen levels (Online Resource 14). Finally, we verified whether ARNT2 binding sites are present within *OLIG2*, *POU3F2* and *SOX9* regulatory regions by ChIP-qPCR experiments using ARNT2 antibodies. This analysis showed an enrichment in ARNT2 binding sites in one or more of the *SOX9*, *POU3F2* and *OLIG2* regulatory regions tested, compared to the house keeping gene *TBP* (Online Resource 15), indicating that *OLIG2*, *POU3F2* and *SOX9* can be directly regulated through ARNT2 binding to their regulatory regions. Altogether these results confirmed the functional relevance of the co-variation of ARNT2 expression with the tumorigenic/stem signature identified by tumor tissue and single cell transcriptome analysis. Our analysis further showed that *ARNT2* down-regulation was accompanied by a decrease in *LEF1* mRNA levels (Fig. 6a, b, Online Resource 13A), whereas the expression of the HIF family members HIF1A, HIF2A and ARNT varied from one cell line to another (Fig. 6a, b, Online Resource 13A).

Taken together, these results demonstrate that *ARNT2* is expressed at the mRNA and protein level within the tumors of patients with glioblastoma. Further, they show that ARNT2 is part of a tumorigenic/stem signature of glioblastoma cells, and regulates the expression of transcription factors previously shown to be involved in the control of glioblastoma cell tumorigenicity.

### ARNT2 is essential for the maintenance of glioblastoma cell tumorigenic properties

The above results, associated with our initial finding of ARNT2 down-regulation in glioblastoma cells deprived of tumorigenic properties, led us to evaluate the role of ARNT2 in the control of glioblastoma cell tumorigenicity using orthotopic xenografts of GBM stem-like cells (6240**, 5706**) expressing either a shControl or a sh*ARNT2*. *ARNT2* knockdown inhibited the proliferation and the clonality of the cells in vitro (Fig. 6c–e, Online Resource 13B and C). Of note, our observation of increased *ARNT* mRNA levels upon *ARNT2* knockdown (Fig. 6a, b) indicates that ARNT cannot compensate for ARNT2 knockdown. Orthotopic xenografts of 1×, 2×, 4× or 14 × 10^4^ 6240** or 5706** GBM stem-like cells stably expressing luciferase and either shControl or sh*ARNT2* were used to follow tumor development with bioluminescent imaging. Results showed a striking reduction in tumor incidence in mice grafted with 6240**- and 5706**-sh*ARNT2* compared to mice grafted with 6240**- and 5706**-shCTL cells (Fig. 6f, i, Online Resource 16A–D). Bioluminescence imaging 42 days post-graft revealed tumor formation in six out of seven, and in six out of six mice engrafted with 14 × 10^4^ 6240**-shControl and 5706**-shControl, respectively (Fig. 6f, i). In contrast, no bioluminescent signal was detected in the mice grafted with 6240**-sh*ARNT2* or 5706**-sh*ARNT2* (Fig. 6f, i). Similar results were obtained when grafting smaller numbers of cells (Online Resource 13). The reduced tumor development in the mice grafted with 6240** and 5706**-sh*ARNT2* was confirmed by immunohistochemistry (Online Resource 16E-F). Survival assays revealed differing long-term consequences of *ARNT2* knockdown according to the GBM stem-like cells grafted. Although we observed a significant improvement in the survival of the mice grafted with either 6240** or 5706** cells expressing sh*ARNT2* (Kaplan–Meier analysis, Fig. 6h, k), only mice grafted with 6240**-sh*ARNT2* eventually developed tumors. Determination of human ARNT2 mRNA levels by QPCR in these tumors showed ARNT2 as well as OLIG2, POU3F2 and SOX9 transcripts levels similar to tumors of the shCTL group (Online Resource 16G). This result indicates that the 6240** cells that formed the tumors escaped ARNT2 inhibition, further pointing to an essential role of this transcription factor for glioblastoma cell aggressiveness. Taken together, these results show that ARNT2 participates in the control of the tumorigenicity of glioblastoma cells.

## Discussion

Understanding the molecular basis of the varying functional cell states that co-exist within glioblastoma and participate in tumor resistance to treatments is of great importance to improve current therapeutic management. Differences in the ability of glioblastoma cells from the same tumor to initiate neoplasms has notably been highlighted by grafting cells sorted from glioblastoma surgical resections in immune-deficient mouse brains [35, 58, 73]. Recent studies have also shown the striking phenotypic plasticity of glioblastoma cells, which can adopt more or less aggressive states during the course of the tumor evolution and treatment [3, 33]. Here, we identified changes in the chromatin state of transcription factors, which accompany the passage of GBM stem-like cells from a highly aggressive to a poorly tumorigenic state. We uncovered a novel transcription factor controlling glioblastoma cell tumorigenicity, which is localized at a node of a transcription factor network controlling glioblastoma cell aggressiveness, and which clusters with a tumorigenic/stem signature of glioblastoma cells at both tissue and single cell levels.

Using the human glioblastoma cell line TG1 expressing or not expressing the micro-RNA cluster miR-302–367 as a model system [22], we profiled histone modifications. The results of our analyses uncovered a subset of genes showing changes in H3K4me3 or H3K27me3 between TG1 cells and TG1-miR cells in which the stem-like and tumorigenic properties have been repressed by expression of the miR-302–367. In agreement with the previously reported association of miR-302–367 with differentiation of GBM stem-like cells [22], [23], ontological pathway analysis of the subset of genes with changes in histone marks showed enrichment in ontological gene groups related to development and engagement in differentiation pathways. Enrichments in terms related to nervous system were also obtained (neuron, neurogenesis, synapse, forebrain), indicating conservation in the tumor cells of an imprint of their tissue of origin. Retrieval of the 202 transcription factors undergoing a change in histone marks further highlighted molecular pathways already identified as important players in the regulation of neural stem/progenitor cell but also of ESC and GBM stem-like cell behaviors, illustrating the pertinence of mapping histone epigenetic marks for identifying regulators of glioblastoma cell properties. For example, we observed an increased H3K27me3 associated with the gene encoding Nanog, a key factor in ESC pluripotency, and which has also been implicated in the maintenance of GBM stem-like cell properties [22, 52, 75]. Similarly, changes were observed for LEF1, an effector of the Wnt signaling pathway known to be involved in neurogenesis [8] and maintenance of GBM stem-like cell [38, 77, 79], TCF3 and TCF7 that are transcriptional regulators of the Wnt pathway in neural stem cells and ESC [40, 74] and the HES bHLH genes and FOXCs transcription factors implicated in the Notch signaling pathway, which is activated in neural stem cells and GBM stem-like cells [36, 72].

Mapping known and predicted protein–protein interactions between transcription factors exhibiting changes in histone marks in GBM stem-like cell lacking tumorigenic properties generated a network articulated around ARNT2. ARNT2, like its paralog ARNT, is considered to act as a dimerization partner of HIF1/2α, the heterodimers triggering the expression of hypoxia-related genes [46, 61]. ARNT2 expression is especially abundant in the central nervous system and kidney, while that of ARNT is ubiquitous [18, 32]. In the central nervous system, *ARNT2* mRNA and protein are enriched in neurons [18]. Although ARNT2/HIFs and ARNT/HIFs heterodimers are equally efficient to ensure neuronal responses to hypoxia [46], ARNT2 protein levels do not increase under hypoxic conditions unlike those of ARNT and HIF1/2α [42, 47]. The role of ARNT2 in cancer is poorly explored. ARNT2 has been associated with increased as well as decreased growth of non-cerebral cancers [39, 43, 46, 48, 59, 60]. In glioblastoma HIF1 and 2α, but not ARNT2, have been associated to adaptation of cancer cells to hypoxic conditions [30, 42]. We found that the profile of *ARNT2* expression in glioblastoma does not correspond with that expected for a hypoxia-related molecule. ARNT2 expression was highest in glioblastoma core zones rather than in the hypoxic necrotic and pseudopalisading zones. This observation favors a hypoxia-independent transcriptional role for ARNT2.

Of note, ARNT2 is one of the few transcription factors switching from the active H3K4me3 to the repressive H3K27me3 mark in GBM stem-like cells expressing miR-302–367. Analysis of publically available data sets indicated that down-regulation of ARNT2 mRNA occurs not only in the TG1-miR cell line, but importantly is also observed in non-tumorigenic glioblastoma cells either directly sorted from patients’ tumors [73] or following serum-induced differentiation of GBM stem-like cell [41]. This was confirmed at the protein level by immunohistochemistry of ARNT2 expression in sub-populations of cells within proliferative zones of patients’ glioblastoma. Furthermore, we demonstrated that *ARNT2* knockdown inhibits tumor-initiating properties in vivo, supporting a role of ARNT2 in the tumorigenicity of glioblastoma cells.

Examination of tumor patients’ transcriptome datasets further associated ARNT2 with a tumorigenic/stem signature of glioblastoma cells [55] at both the tissue and single cell levels. To ascertain the functional relevance of this association, we focused on three transcription factors members of this stem signature *SOX9*, *POU3F2* and *OLIG2,* since the knockdown of these factors has previously been reported to inhibit glioblastoma cell tumorigenicity in vivo [31, 44, 68]. We found that *ARNT2* knockdown not only impaired the cell tumorigenicity in vivo but also resulted in decreased expression of *SOX9*, *POU3F2* and *OLIG2,* hence placing ARNT2 at the core of transcriptional regulations of glioblastoma cell tumorigenicity.

In conclusion, our results uncover a novel transcription factor essential for glioblastoma cell tumorigenic properties, show its functional relevance within the context of the patients’ tumor, and shed new lights on the combinatorial organization of the transcription factor networks that regulate glioblastoma cell aggressiveness.

## Electronic supplementary material

Below is the link to the electronic supplementary material.

Supplementary material 1 (XLSX 59 kb)

Supplementary material 2 (XLSX 9 kb)

Supplementary material 3 (XLSX 208 kb)

Supplementary material 4 (PDF 344 kb)

Supplementary material 5 (XLSX 22 kb)

Supplementary material 6 (XLSX 12 kb)

Supplementary material 7 (PDF 159 kb)

Supplementary material 8 (PDF 258 kb)

Supplementary material 9 (PDF 11,009 kb)

Supplementary material 10 (XLSX 9 kb)

Supplementary material 11 (PDF 258 kb)

Supplementary material 12 (PDF 215 kb)

Supplementary material 13 (PDF 346 kb)

Supplementary material 14 (PDF 158 kb)

Supplementary material 15 (PDF 410 kb)

Supplementary material 16 (PDF 11,179 kb)
