# Supplementary material for: Changes in chromatin state reveal ARNT2 at a node of a tumorigenic transcription factor signature driving glioblastoma cell aggressiveness
Source: Acta Neuropathol. 2017 Nov 17;135(2):267–83. doi: 10.1007/s00401-017-1783-x (PMC5773658; doi:10.1007/s00401-017-1783-x)
Supplement: Supplementary file 4 — Supplementary material 4 (PDF 344 kb) [file 401_2017_1783_MOESM4_ESM.pdf]

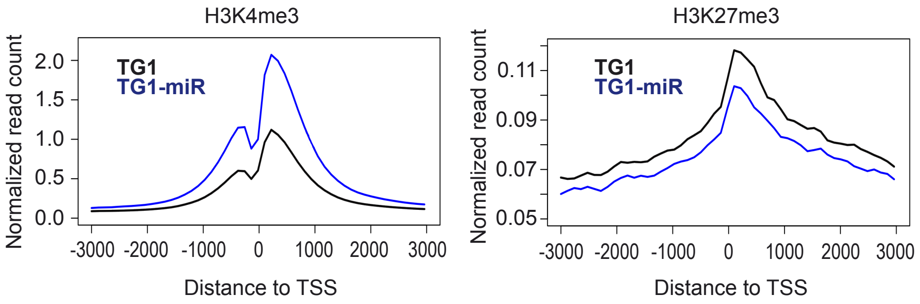

Online Resource 4. Classical distribution of H3K4me3 and H3K27me3 marks around transcriptional start sites (TSS) in TG1 and TG1-miR. H3K4me3 is concentrated around the TSS (left panel). H3K27me3 is both enriched at the TSS and along the gene body (right panel). Profiles were established from the set of genes associated with H3K4me3 or H3K27me3 marks. Normalized read counts (y-axis) correspond to the number of sequences identified by mega base per million of mapped sequences.

Changes in chromatin state reveal ARNT2 at a node of a tumorigenic transcription factor signature driving glioblastoma cell aggressiveness.

A. Bogeas, G. Morvan-Dubois, E. A. El-Habr, F-X. Lejeune, M. Defrance, A. Narayanan, K. Kuranda, F. Burel-Vandenbos, S. Sayd, V. Delaunay, L. G. Dubois, H. Parrinello, S. Rialle, S. Fabrega, A. Ibdaih, J. Haiech, I. Bièche, T. Virolle, M. Goodhardt, H. Chneiweiss,

Corresponding authors : herve.chneiweiss@inserm.fr; marie-pierre.junier@inserm.fr

M-P. Junier
Acta Neuropathologica
