# Supplementary material for: Changes in chromatin state reveal ARNT2 at a node of a tumorigenic transcription factor signature driving glioblastoma cell aggressiveness
Source: Acta Neuropathol. 2017 Nov 17;135(2):267–83. doi: 10.1007/s00401-017-1783-x (PMC5773658; doi:10.1007/s00401-017-1783-x)
Supplement: Supplementary file 7 — Supplementary material 7 (PDF 159 kb) [file 401_2017_1783_MOESM7_ESM.pdf]

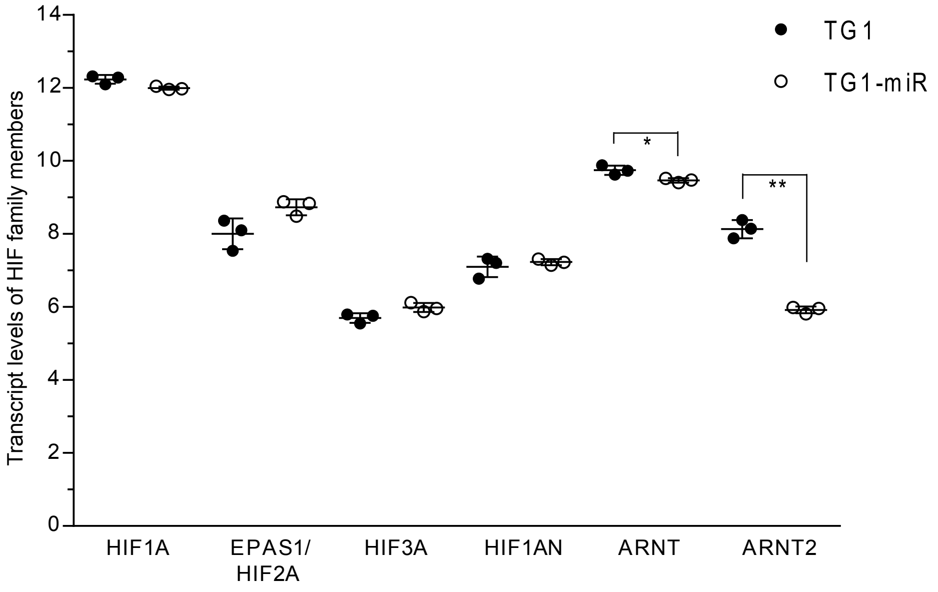

Online Resource 7. HIF family members mRNA levels in TG1 and TG1-miR. Note the robust decrease in ARNT2 mRNA levels following miR-302-367 expression. Microarray transcriptome analysis. **p<0.01, *p<0.05, unpaired t test with Welch's correction, mean ± SD, n=3 independent biological samples.

Changes in chromatin state reveal ARNT2 at a node of a tumorigenic transcription factor signature driving glioblastoma cell aggressiveness.

A. Bogeas, G. Morvan-Dubois, E. A. El-Habr, F-X. Lejeune, M. Defrance, A. Narayanan, K. Kuranda, F. Burel-Vandenbos, S. Sayd, V. Delaunay, L. G. Dubois, H. Parrinello, S. Rialle, S. Fabrega, A. Ibdaih, J. Haiech, I. Bièche, T. Virolle, M. Goodhardt, H. Chneiweiss, M-P. Junier

Acta Neuropathologica

Corresponding authors : herve.chneiweiss@inserm.fr; marie-pierre.junier@inserm.fr
