# Supplementary material for: Changes in chromatin state reveal ARNT2 at a node of a tumorigenic transcription factor signature driving glioblastoma cell aggressiveness
Source: Acta Neuropathol. 2017 Nov 17;135(2):267–83. doi: 10.1007/s00401-017-1783-x (PMC5773658; doi:10.1007/s00401-017-1783-x)
Supplement: Supplementary file 8 — Supplementary material 8 (PDF 258 kb) [file 401_2017_1783_MOESM8_ESM.pdf]

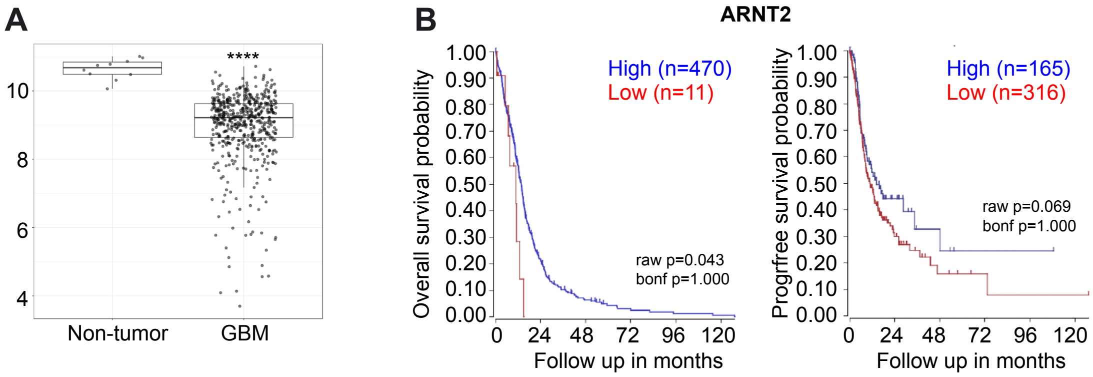

Online Resource 8. ARNT2 mRNA levels in patients' glioblastoma.

A. ARNT2 mRNA levels in glioblastoma compared to non-tumor brain tissues Analysis performed on the GlioVis platform (http://gliovis.bioinfo.cnio.es/) using transcriptomes of 481 untreated primary human glioblastoma of the TCGA dataset, and 10 brain tissue samples. **** p<0.0001, pairwise t test.

B. ARNT2 mRNA levels do not discriminate patients according to overall survival or progression free survival. Analysis Cut-off values were determined by automatic scanning of the entire set of values (transcriptomes of 481 untreated primary human glioblastoma of the TCGA dataset) using the R2 Genomics Analysis and Visualization Platform (http://r2.amc.nl).

Changes in chromatin state reveal ARNT2 at a node of a tumorigenic transcription factor signature driving glioblastoma cell aggressiveness.

A. Bogeas, G. Morvan-Dubois, E. A. El-Habr, F-X. Lejeune, M. Defrance, A. Narayanan, K. Kuranda, F. Burel-Vandenbos, S. Sayd, V. Delaunay, L. G. Dubois, H. Parrinello, S. Rialle, S. Fabrega, A. Ibdaih, J. Haiech, I. Bièche, T. Virolle, M. Goodhardt, H. Chneiweiss, M-P. Junier

Acta Neuropathologica

Corresponding authors : herve.chneiweiss@inserm.fr; marie-pierre.junier@inserm.fr
