# Supplementary material for: Changes in chromatin state reveal ARNT2 at a node of a tumorigenic transcription factor signature driving glioblastoma cell aggressiveness
Source: Acta Neuropathol. 2017 Nov 17;135(2):267–83. doi: 10.1007/s00401-017-1783-x (PMC5773658; doi:10.1007/s00401-017-1783-x)
Supplement: Supplementary file 9 — Supplementary material 9 (PDF 11,009 kb) [file 401_2017_1783_MOESM9_ESM.pdf]

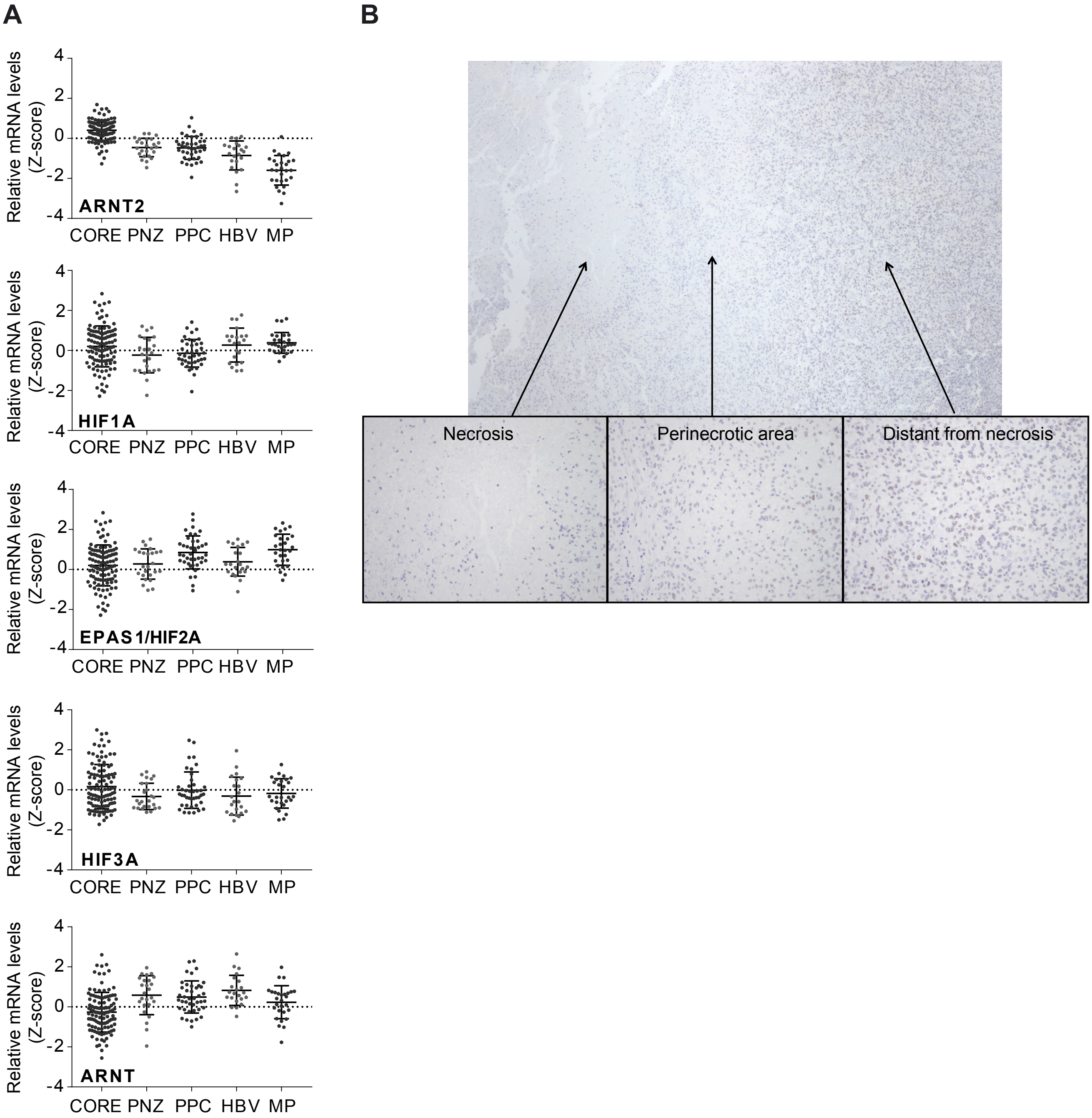

Online Resource 9. Comparison of relative mRNA levels of HIF family members across distinct glioblastoma zones.
A. Z score from IVY dataset (glioblastoma.alleninstitute.org). CORE: core zone of glioblastoma. PNZ: perinecrotic zone. PPC: pseudopalisading cells. HBV: hyperplastic blood vessels. MP: microvascular proliferation.
B. Increased ARNT2
expression with distance from necrosis in patient's glioblastoma, as shown with ARNT2 immunostochemical staining.

Changes in chromatin state reveal ARNT2 at a node of a tumorigenic transcription factor signature driving glioblastoma cell aggressiveness.

A. Bogeas, G. Morvan-Dubois, E. A. El-Habr, F-X. Lejeune, M. Defrance, A. Narayanan, K. Kuranda, F. Burel-Vandenbos, S. Sayd, V. Delaunay, L. G. Dubois, H. Parrinello, S. Rialle, S. Fabrega, A. Ibdaih, J. Haiech, I. Bièche, T. Virolle, M. Goodhardt, H. Chneiweiss, M-P. Junier

Acta Neuropathologica

Corresponding authors : herve.chneiweiss@inserm.fr; marie-pierre.junier@inserm.fr
