# Supplementary material for: Changes in chromatin state reveal ARNT2 at a node of a tumorigenic transcription factor signature driving glioblastoma cell aggressiveness
Source: Acta Neuropathol. 2017 Nov 17;135(2):267–83. doi: 10.1007/s00401-017-1783-x (PMC5773658; doi:10.1007/s00401-017-1783-x)
Supplement: Supplementary file 11 — Supplementary material 11 (PDF 258 kb) [file 401_2017_1783_MOESM11_ESM.pdf]

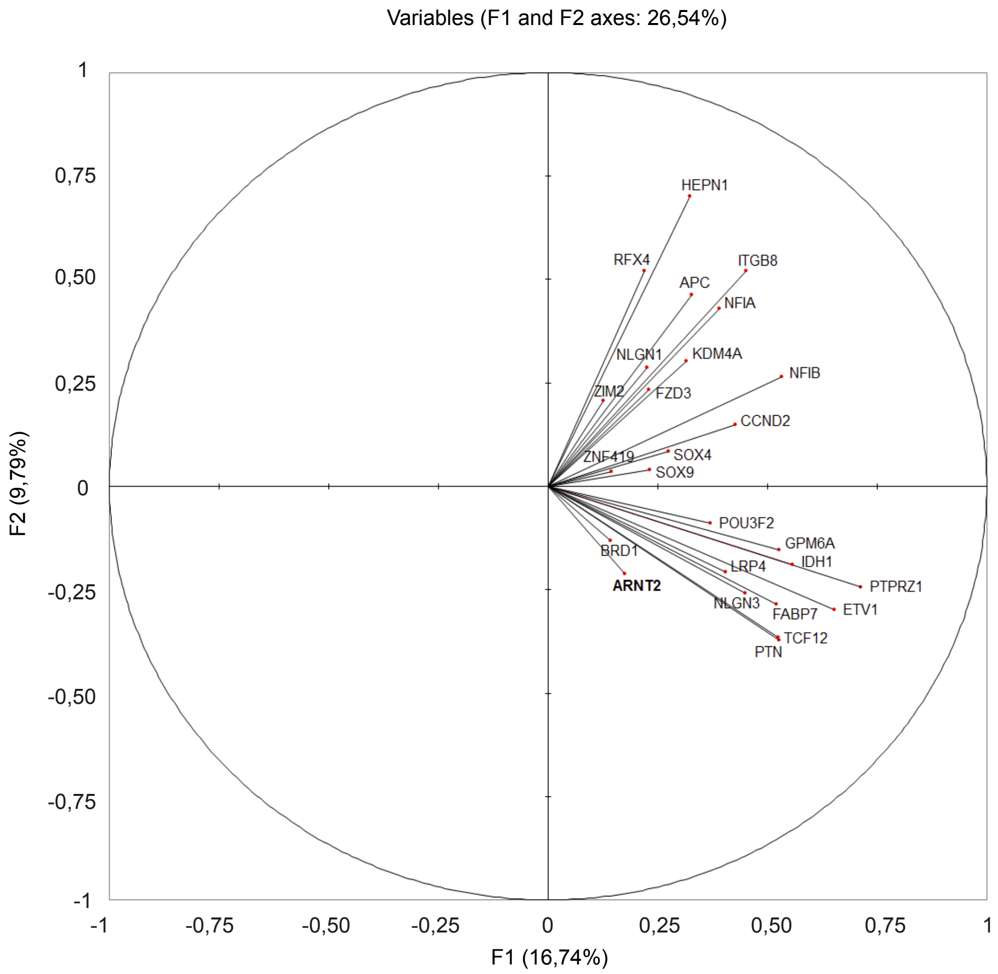

Online Resource 11. ARNT2 expression correlates with the tumorigenic/stem signature of glioblastoma cells at the single-cell level.

Correlation circle from PCA analysis showing stem signature gene expression correlation with F1 and F2 principal components. Analysis performed with transcriptome data from Patel and colleagues (Patel et al., 2014), which gathers transcriptome profiles derived from RNA-seq of 254 cells sampled from 5 distinct patients' glioblastoma.

See Online Resource 10 for the list of the signature's components.

Changes in chromatin state reveal ARNT2 at a node of a tumorigenic transcription factor signature driving glioblastoma cell aggressiveness.

A. Bogeas, G. Morvan-Dubois, E. A. El-Habr, F-X. Lejeune, M. Defrance, A. Narayanan, K. Kuranda, F. Burel-Vandenbos, S. Sayd, V. Delaunay, L. G. Dubois, H. Parrinello, S. Rialle, S. Fabrega, A. Ibdaih, J. Haiech, I. Bièche, T. Virolle, M. Goodhardt, H. Chneiweiss, M-P. Junier

Acta Neuropathologica

Corresponding authors : herve.chneiweiss@inserm.fr; marie-pierre.junier@inserm.fr
