# Supplementary material for: Changes in chromatin state reveal ARNT2 at a node of a tumorigenic transcription factor signature driving glioblastoma cell aggressiveness
Source: Acta Neuropathol. 2017 Nov 17;135(2):267–83. doi: 10.1007/s00401-017-1783-x (PMC5773658; doi:10.1007/s00401-017-1783-x)
Supplement: Supplementary file 12 — Supplementary material 12 (PDF 215 kb) [file 401_2017_1783_MOESM12_ESM.pdf]

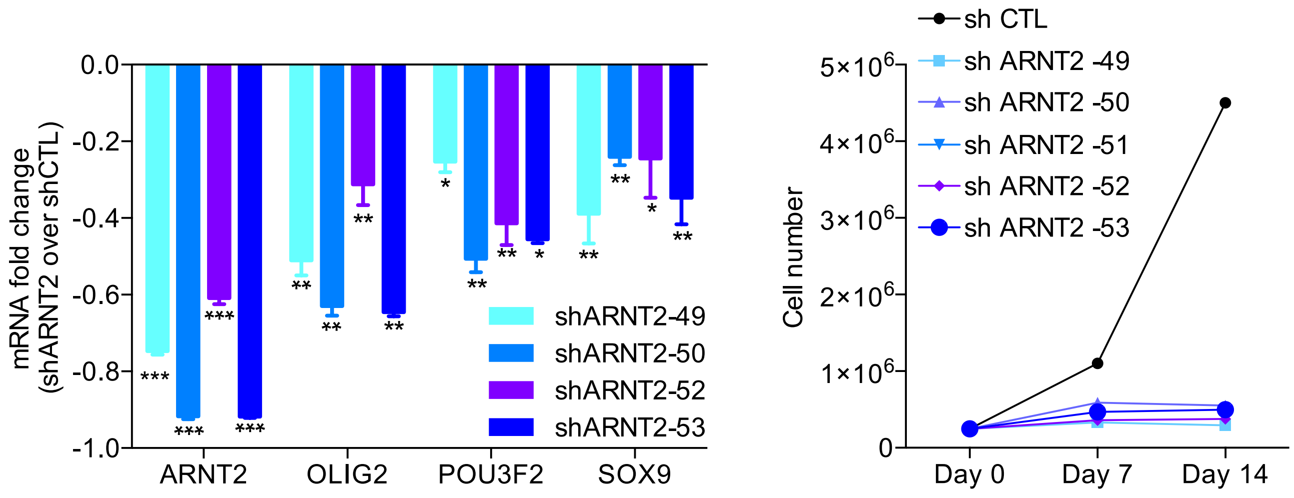

Online Resource 12. Comparative effect of distinct shRNAs targeting ARNT2 on ARNT2, OLIG2, POU3F2 and SOX9 mRNA levels (left panel), and on cell proliferation (right panel). 6240** GBM stem-like cells. **p<0.05, **p<0.01, ***p<0.001, unpaired t test with Welch's correction,mean±SD, n=4. shARNT2-53 was selected for further experiments and is hence after designed as shARNT2.

Changes in chromatin state reveal ARNT2 at a node of a tumorigenic transcription factor signature driving glioblastoma cell aggressiveness.

A. Bogeas, G. Morvan-Dubois, E. A. El-Habr, F-X. Lejeune, M. Defrance, A. Narayanan, K. Kuranda, F. Burel-Vandenbos, S. Sayd, V. Delaunay, L. G. Dubois, H. Parrinello, S. Rialle, S. Fabrega, A. Ibdaih, J. Haiech, I. Bièche, T. Virolle, M. Goodhardt, H. Chneiweiss, M-P. Junier

Acta Neuropathologica

Corresponding authors : herve.chneiweiss@inserm.fr; marie-pierre.junier@inserm.fr
