# Supplementary material for: Changes in chromatin state reveal ARNT2 at a node of a tumorigenic transcription factor signature driving glioblastoma cell aggressiveness
Source: Acta Neuropathol. 2017 Nov 17;135(2):267–83. doi: 10.1007/s00401-017-1783-x (PMC5773658; doi:10.1007/s00401-017-1783-x)
Supplement: Supplementary file 13 — Supplementary material 13 (PDF 346 kb) [file 401_2017_1783_MOESM13_ESM.pdf]

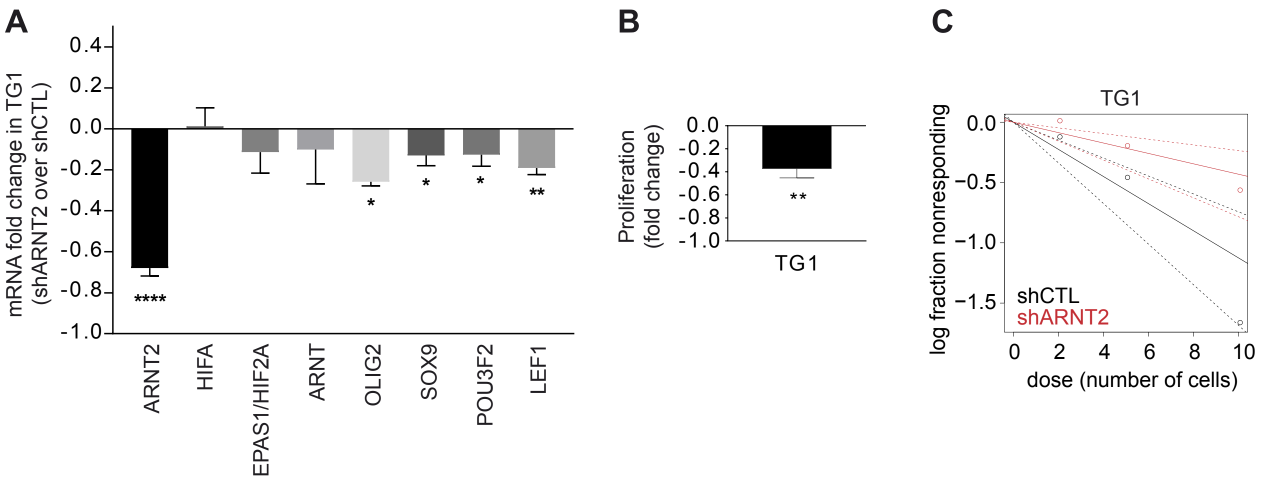

Online Resource 13. Responses of TG1 GBM stem-like cells to ARNT2 down-regulation.
A. Consequences of ARNT2 down-regulation on the expression of HIF family members (HIF1A, HIF2A, ARNT), on components of the tumorigenic signature of glioblastoma cells (OLIG2, SOX9, POU3F2), and on the effector of the Wnt-signaling pathway, LEF1. QPCR assay. Results are presented as fold changes in mRNA levels detected in cells expressing shARNT2 compared to shControl (shCTL). *p<0.05, **p<0.01, ****p<0.0001, unpaired t-test with Welch's correction, mean ± SD, n=3.
B. Down-regulation of ARNT2 is accompanied with decreased cell proliferation. shARNT2 versus shControl. **p<0.01, unpaired t-test with Welch's correction, mean ± SD, n=3 independent biological samples.
C. Knocking down ARNT2 impairs the sphere-forming capability of TG1. Extreme limiting dilution assays. Sphere formation was scored 7 days after seeding. Frequency of sphere-forming cells: TG1 shCTL = 1/8.88 (lower 13.4, upper 5.93); TG1 shARNT2 1/23.17 (lower 42.5, upper 12.75), n = 16, p = 8.12 10-3.

Changes in chromatin state reveal ARNT2 at a node of a tumorigenic transcription factor signature driving glioblastoma cell aggressiveness.
A. Bogeas, G. Morvan-Dubois, E. A. El-Habr, F-X. Lejeune, M. Defrance, A. Narayanan, K. Kuranda, F. Burel-Vandenbos, S. Sayd, V. Delaunay, L. G. Dubois, H. Parrinello, S. Rialle, S. Fabrega, A. Ibdaih, J. Haiech, I. Bièche, T. Virolle, M. Goodhardt, H. Chneiweiss, M-P. Junier
Acta Neuropathologica
Corresponding authors : herve.chneiweiss@inserm.fr; marie-pierre.junier@inserm.fr
