# Supplementary material for: Changes in chromatin state reveal ARNT2 at a node of a tumorigenic transcription factor signature driving glioblastoma cell aggressiveness
Source: Acta Neuropathol. 2017 Nov 17;135(2):267–83. doi: 10.1007/s00401-017-1783-x (PMC5773658; doi:10.1007/s00401-017-1783-x)
Supplement: Supplementary file 14 — Supplementary material 14 (PDF 158 kb) [file 401_2017_1783_MOESM14_ESM.pdf]

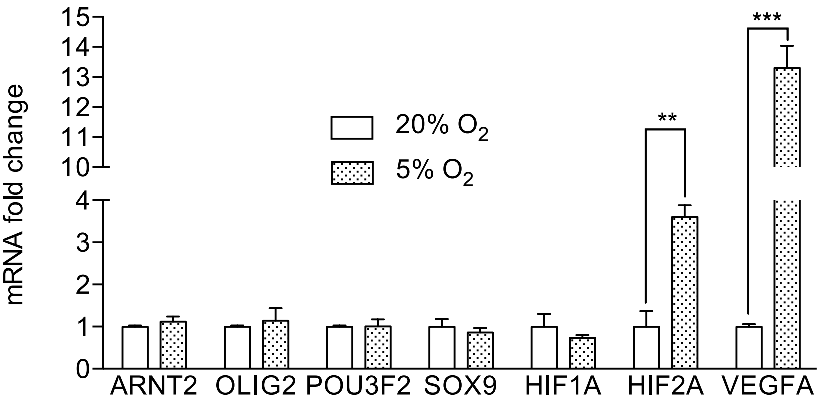

Online Resource 14. Reduction in oxygen levels from 20 to 5% induces increase in HIF2A and VEGF2 mRNA levels without affecting ARNT2, OLIG2, POU3F2 and SOX9 mRNA levels. 5706** Mean±SD, n=3.

Changes in chromatin state reveal ARNT2 at a node of a tumorigenic transcription factor signature driving glioblastoma cell aggressiveness.

A. Bogeas, G. Morvan-Dubois, E. A. El-Habr, F-X. Lejeune, M. Defrance, A. Narayanan, K. Kuranda, F. Burel-Vandenbos, S. Sayd, V. Delaunay, L. G. Dubois, H. Parrinello, S. Rialle, S. Fabrega, A. Ibdaih, J. Haiech, I. Bièche, T. Virolle, M. Goodhardt, H. Chneiweiss, M-P. Junier

Acta Neuropathologica

Corresponding authors : herve.chneiweiss@inserm.fr; marie-pierre.junier@inserm.fr
