# Supplementary material for: Changes in chromatin state reveal ARNT2 at a node of a tumorigenic transcription factor signature driving glioblastoma cell aggressiveness
Source: Acta Neuropathol. 2017 Nov 17;135(2):267–83. doi: 10.1007/s00401-017-1783-x (PMC5773658; doi:10.1007/s00401-017-1783-x)
Supplement: Supplementary file 15 — Supplementary material 15 (PDF 410 kb) [file 401_2017_1783_MOESM15_ESM.pdf]

A

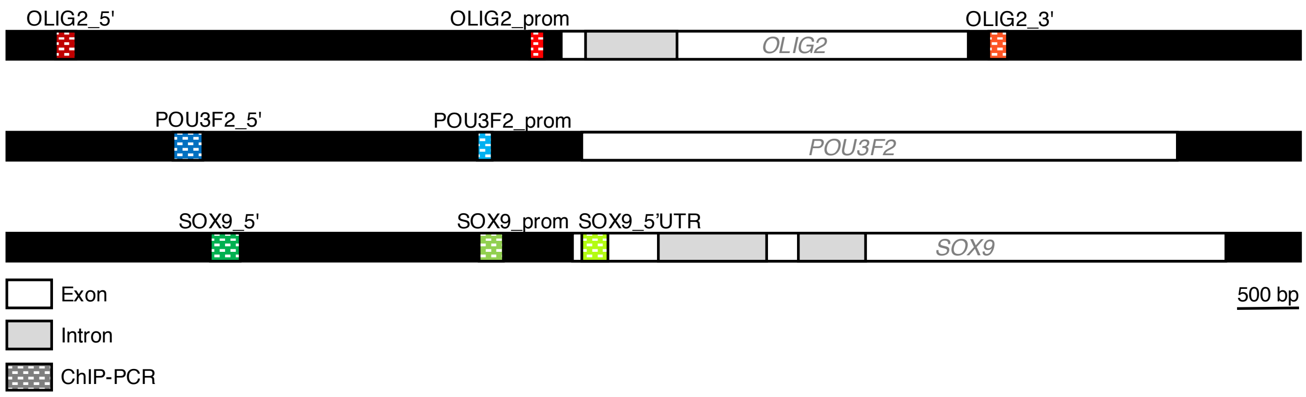

B

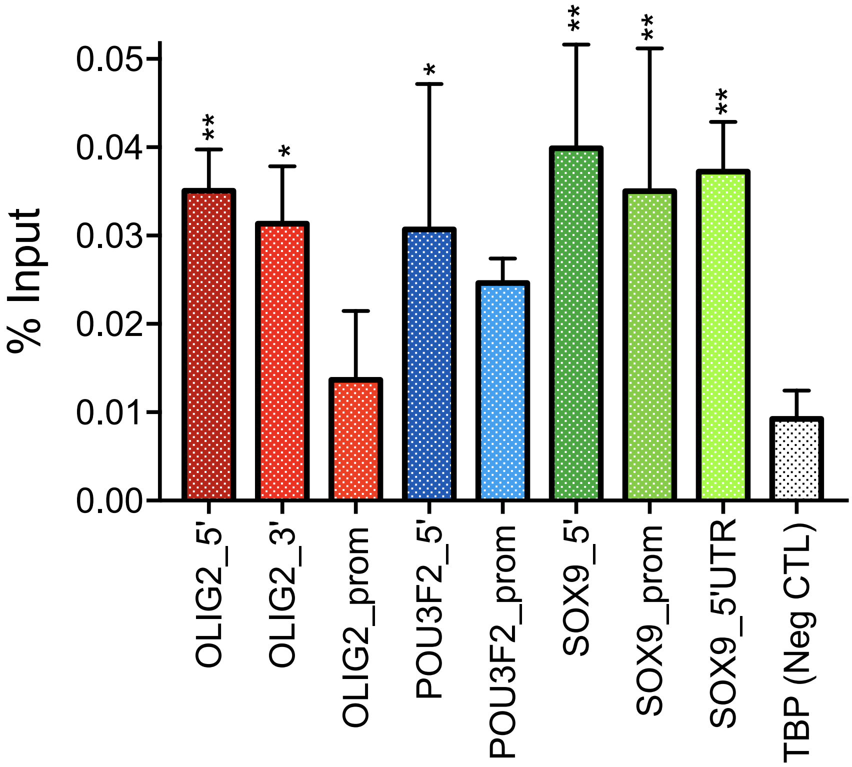

Online Resource 15. **OLIG2, POU3F2 and SOX9 gene loci contain ARNT2 binding sites.**
A. Localization of the primers used in ARNT2-ChIP-QPCR, over the OLIG2, POU3F2 and SOX9 gene loci. All primers are localized in potential regulatory regions in a +/- 10kb window around the SOX9, POU3F2 and OLIG2 coding sequences using published ChIP data performed with antibodies directed against H3K4ac (histone 3 acetylated on lysine 4), known to be enriched in regulatory regions (NCBI, SRX114494 SRA data). Primers were designed to allow amplification of 100 to 250 bp sequences of each region of interest.
B. QPCR results showing enrichment in ARNT2 binding sites in OLIG2 3' and 5' region, POU3F2 5' region, SOX9 5'UTR, promoter and first exon region, compared to TBP negative control. Mean ± SD, n=3. * $p<0.05$, ** $p<0.01$, One way ANOVA.

Changes in chromatin state reveal ARNT2 at a node of a tumorigenic transcription factor signature driving glioblastoma cell aggressiveness.
A. Bogeas, G. Morvan-Dubois, E. A. El-Habr, F-X. Lejeune, M. Defrance, A. Narayanan, K. Kuranda, F. Burel-Vandenbos, S. Sayd, V. Delaunay, L. G. Dubois, H. Parrinello, S. Rialle, S. Fabrega, A. Ibdaih, J. Haiech, I. Bièche, T. Virolle, M. Goodhardt, H. Chneiweiss, M-P. Junier
Acta Neuropathologica
Corresponding authors : herve.chneiweiss@inserm.fr; marie-pierre.junier@inserm.fr
