# Supplementary material for: Changes in chromatin state reveal ARNT2 at a node of a tumorigenic transcription factor signature driving glioblastoma cell aggressiveness
Source: Acta Neuropathol. 2017 Nov 17;135(2):267–83. doi: 10.1007/s00401-017-1783-x (PMC5773658; doi:10.1007/s00401-017-1783-x)
Supplement: Supplementary file 16 — Supplementary material 16 (PDF 11,179 kb) [file 401_2017_1783_MOESM16_ESM.pdf]

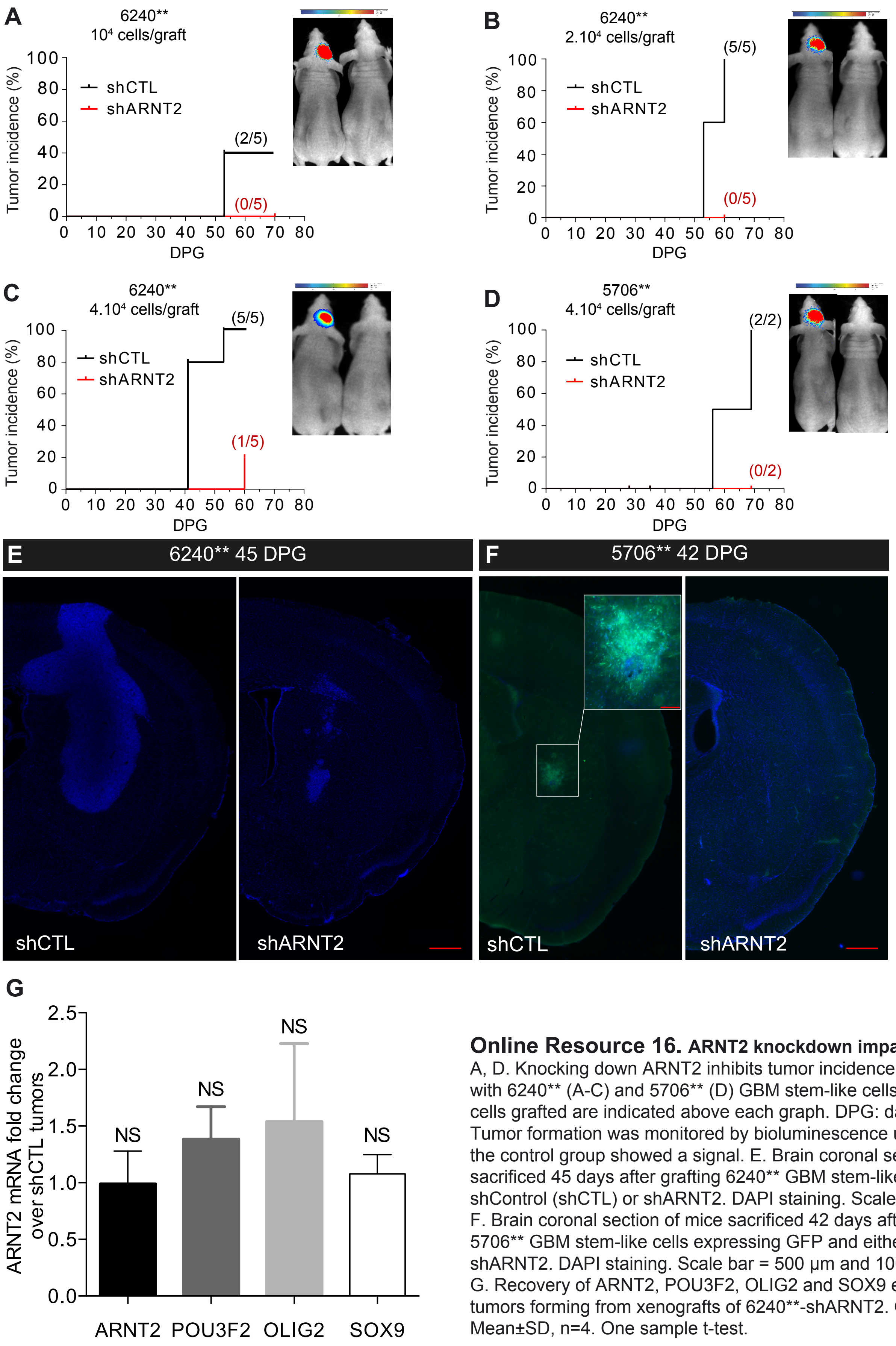

Online Resource 16. **ARNT2 knockdown impairs tumor initiation.** A, D. Knocking down ARNT2 inhibits tumor incidence in mice grafted with 6240** (A-C) and 5706** (D) GBM stem-like cells. The numbers of cells grafted are indicated above each graph. DPG: days post-graft. Tumor formation was monitored by bioluminescence until all mice of the control group showed a signal. E. Brain coronal section of mice sacrificed 45 days after grafting 6240** GBM stem-like cells expressing shControl (shCTL) or shARNT2. DAPI staining. Scale bar = 500 µm. F. Brain coronal section of mice sacrificed 42 days after grafting 5706** GBM stem-like cells expressing GFP and either shControl or shARNT2. DAPI staining. Scale bar = 500 µm and 100 µm in the inset. G. Recovery of ARNT2, POU3F2, OLIG2 and SOX9 expressions in tumors forming from xenografts of 6240**-shARNT2. QPCR assay. Mean±SD, n=4. One sample t-test.

Changes in chromatin state reveal ARNT2 at a node of a tumorigenic transcription factor signature driving glioblastoma cell aggressiveness.

A. Bogeas, G. Morvan-Dubois, E. A. El-Habr, F-X. Lejeune, M. Defrance, A. Narayanan, K. Kuranda, F. Burel-Vandenbos, S. Sayd, V. Delaunay, L. G. Dubois, H. Parrinello, S. Rialle, S. Fabrega, A. Ibdaih, J. Haiech, I. Bièche, T. Virolle, M. Goodhardt, H. Chneiweiss, M-P. Junier

Acta Neuropathologica

Corresponding authors : herve.chneiweiss@inserm.fr; marie-pierre.junier@inserm.fr
